# Construction and Validation of a Robust Cancer Stem Cell-Associated Gene Set-Based Signature to Predict Early Biochemical Recurrence in Prostate Cancer

**DOI:** 10.1155/2020/8860788

**Published:** 2020-10-09

**Authors:** Bide Liu, Xun Li, Jiuzhi Li, Hongyong Jin, Hongliang Jia, Xiaohu Ge

**Affiliations:** ^1^Xinjiang Medical University, No. 393 Xinyi Road, Urumqi, Xinjiang 830011, China; ^2^Laboratory of Urology, People's Hospital of Xinjiang Uygur Autonomous Region, No. 91 Tianchi Road, Urumqi, Xinjiang 830001, China; ^3^Department of Urology, People's Hospital of Xinjiang Uygur Autonomous Region, No. 91 Tianchi Road, Urumqi, Xinjiang 830001, China; ^4^Department of Vascular Surgery, People's Hospital of Xinjiang Uygur Autonomous Region, No. 91 Tianchi Road, Urumqi, Xinjiang 830001, China

## Abstract

**Background:**

Postoperative early biochemical recurrence (BCR) was an essential indicator for recurrence and distant metastasis of prostate cancer (PCa). The aim of this study was to construct a cancer stem cell- (CSC-) associated gene set-based signature to identify a subgroup of PCa patients who are at high risk of early BCR.

**Methods:**

The PCa dataset from The Cancer Genome Atlas (TCGA) was randomly separated into discovery and validation set. Patients in discovery set were divided into early BCR group and long-term survival group. Propensity score matching analysis and differentially expressed gene selection were used to identify candidate CSC-associated genes. The LASSO Cox regression model was finally performed to filter the most useful prognostic CSC-associated genes for predicting early BCR.

**Results:**

By applying the LASSO Cox regression model, we built a thirteen-CSC-associated gene-based early BCR-predicting signature. In the discovery set, patients in high-risk group showed significantly poorer BCR free survival than that patients in low-risk group (HR: 4.91, 95% CI: 2.75–8.76, *P* < 0.001). The results were further validated in the internal validation set (HR: 2.99, 95% CI: 1.34–6.70, *P* = 0.005). Time-dependent ROC at 1 year suggested that the CSC gene signature (AUC = 0.800) possessed better predictive value than any other clinicopathological features in the entire TCGA cohort. Additionally, survival decision curve analysis revealed a considerable clinical usefulness of the CSC gene signature.

**Conclusions:**

We successfully developed a CSC-associated gene set-based signature that can accurately predict early BCR in PCa cancer.

## 1. Introduction

Radical surgery followed by radiation therapy has been demonstrated to be able to improve the prognosis of localized prostate cancer (PCa) [1]. However, there are still about 20% of these patients will develop biochemical recurrence (BCR) which has been confirmed as an essential early indication of cancer-specific survival [2]. BCR was defined as two or more consecutive PSA values of >0.20 ng/mL. The recurrence occurring within two years after surgery was usually considered as early BCR [3]. Those patients who developed earlier BCR were trended to have poorer prognosis [4]. Consequently, it is crucial to identify and detect early BCR in PCa.

Previous studies have identified several clinicopathological factors including Gleason score, TNM stage, and margin status as predictive variables for BCR [5, 6]. However, for the great heterogeneity of PCa, prognosis varies significantly in PCa patients with same tumor stage and similar clinicopathological features [7, 8]. Therefore, more ideal biomarker and predictive model are warranted to be developed. Recent studies in many malignancies, including PCa, suggested that multigene classifier or gene signature can make a good prediction of tumor prognosis [9–11]. However, limited robust signature has been constructed to predict early BCR [12, 13]. Searching a more valuable signaling pathway- or multiple signaling pathway-based signature might be of concrete predictive value in the detection of PCa patients with early BCR.

Cancer stem cells (CSCs) have been established as a critical component of tumor that may trigger tumorigenesis, self-renewal, differentiation, and resistance to therapy in PCa [14, 15]. Previous basic researches have demonstrated that CSCs have the radical ability to activate several highly conserved signaling pathways including the Notch [16], Hedgehog [17], and Wnt pathways [18] that are related to tissue development and homeostasis. These signaling pathways have complex crosstalk and function great role in tumorigenesis and tumor progression. We hypothesized that the development of a CSC-associated gene set-based signature will be of great significance to predict early BCR in PCa.

In this study, CSC signaling pathways and the gene expression profiles of 418 PCa patients form The Cancer Genome Atlas (TCGA) were analyzed. By using the sample-splitting method and Cox regression analysis, we constructed a prognostic thirteen-CSC-associated gene-based signature with high predictive ability. This CSC-related gene set signature will help to individualize postoperative treatment and follow-up scheme for PCa patients.

## 2. Methods

### 2.1. Data Source

All the data used in this study was derived from TCGA database. The integrated mRNA expression (Fragments Per Kilobase Of Exon Per Million Fragments Mapped, FPKM) and clinical data were downloaded from the Cancer Genomics Browser (https://genome-cancer.ucsc.edu). A total of 418 PCa patients with complete information of BCR and basic clinicopathological features were identified in this study. In this cohort, 92 patients developed BCR and 42 (45.7%) patients developed BCR within 1 year.

### 2.2. Candidate CSC-Associated Genes

All the genes that are associated with the signaling or transcriptional regulators of CSC in the signaling pathways of Hedgehog, Notch, and Wnt were selected into this study. The candidate genes were based on polymerase chain reaction array gene lists from SABiosciences (http://www.sabiosciences.com/PCRArrayPlate.php) [19]. The identified gene list consists of Hedgehog/Notch/Wnt ligands, receptors and regulators, and their downstream target proteins [19].

### 2.3. Identification of Prognostic CSC-Associated Genes

Patients in TCGA database were randomly divided into discovery and validation sets at a ratio of 1/4. 313 (75%) patients were assigned to the discovery series, and 105 (25%) were assigned to the validation series. Early BCR was defined as the BCR occurring within 1 year after initial primary resection. Patients in discovery set TCGA were identified and separated into early BCR group and long-term survival group (no BCR after a minimum of 5-year follow-up). Propensity score (PS) matching analysis was then conducted between the two groups adjusting for Gleason score, T stage, and N stage. Matched ratio was set as 1 : 1. Finally, twenty-three paired patients in the discovery series were selected to perform differential expression analysis among CSC-associated genes. The Linear Models for Microarray data (LIMMA) method was used to generate the differentially expressed genes between early BCR and long-term survival patients. Lastly, the LASSO Cox regression model was performed to select the most critical CSC-related genes that associated with early BCR.

### 2.4. Development of CSC-Based Risk Score and Statistical Analysis

By performing LASSO Cox regression model, we identified a list of CSC-associated genes and constructed a multigene-based signature to predict the early BCR of PCa patients in the discovery set. A standard risk score calculation formula was used to generate the risk score of each patient by combining the expression value of the genes and the corresponding LASSO Cox regression coefficients. Patients were divided into high-risk and low-risk of BCR groups based on the median risk score determined in the discovery set.

Kaplan Meier survival analysis was used to compare the differences between high- and low-risk group. Time-dependent receiver operating characteristic (ROC) analysis was performed, and area under the curve (AUC) was used to indicate the prognostic accuracy of clinical feature and multigene signature. Decision curve analysis (DCA) examined the theoretical relationship between the threshold survival probability at 1 year of PCa and the relative value of false-positive and false-negative results to determine the clinical utility of the signature. Harrell's concordance index (c-index) was used to test and discriminative ability of the developed nomogram model. All statistical analyses were performed using of R (version 3.5.0, http://www.r-project.org). All statistical tests were 2-sided, and *P* value < 0.05 were considered statistically significant.

## 3. Results

### 3.1. Development of CSC Gene-Based Signature from Discovery Series

**Figure 1:**
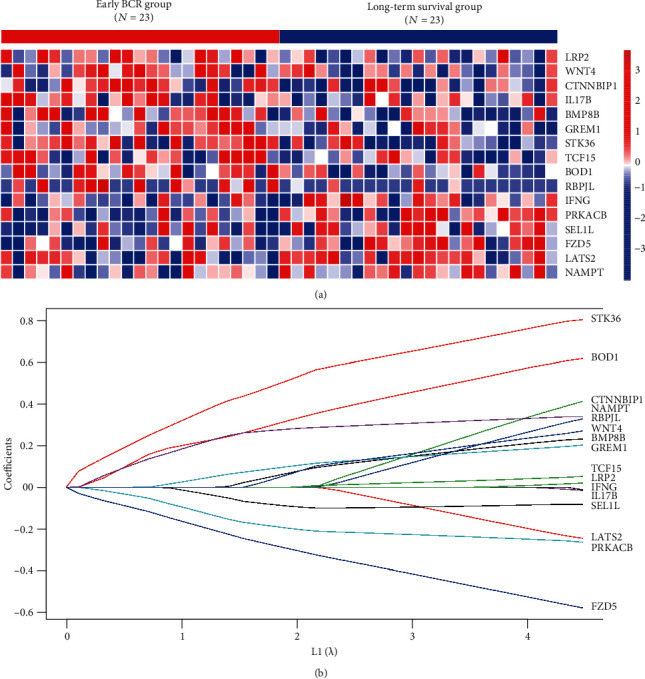
(a) Heat map showed sixteen differentially expressed CSC-associated genes in PCa between early BCR and long-term survival group in discover set. (b) LASSO coefficient profiles of the sixteen early BCR-associated CSC genes.

Before PS matching analysis, patients in early BCR group have significantly higher Gleason score, T stage, and N stage (Table 1). After PS matching, no significant differences were observed between the two risk groups among age, Gleason score, and tumor stage variables (Table 1). Differential gene expression analysis was then performed and found that sixteen CSC-associated genes were significantly differentially expressed (*P* < 0.05, Figure 1(a)). LASSO coefficient profiles of the sixteen genes were shown in Figure 1(b). The detailed information of fold change and significance of these sixteen genes are shown in Table S1. At 10-fold cross-validation, the minimized *λ* method resulted in thirteen prognostic CSC-associated genes. Risk score was lastly calculated based on the expression value of the thirteen genes and risk regression coefficients for each patient: Risk score = (0.245 × expression level of BMP8B) + (0.630 × expression level of BOD1) + (0.446 × expression level of CTNNBIP1) + (−0.594 × expression level of FZD5) + (0.207 × expression level of GREM1) + (−0.265 × expression level of LATS2) + (0.349 × expression level of NAMPT) + (−0.263 × expression level of PRKACB) + (0.342 × expression level of RBPJL) + (−0.076 × expression level of SEL1L) + (0.825 × expression level of STK36) + (0.05 × expression level of TCF15) + (0.287 × expression level of WNT4).

### 3.2. Prognostic Value of CSC Gene-Based Signature in Training and Validation Sets

**Figure 2:**
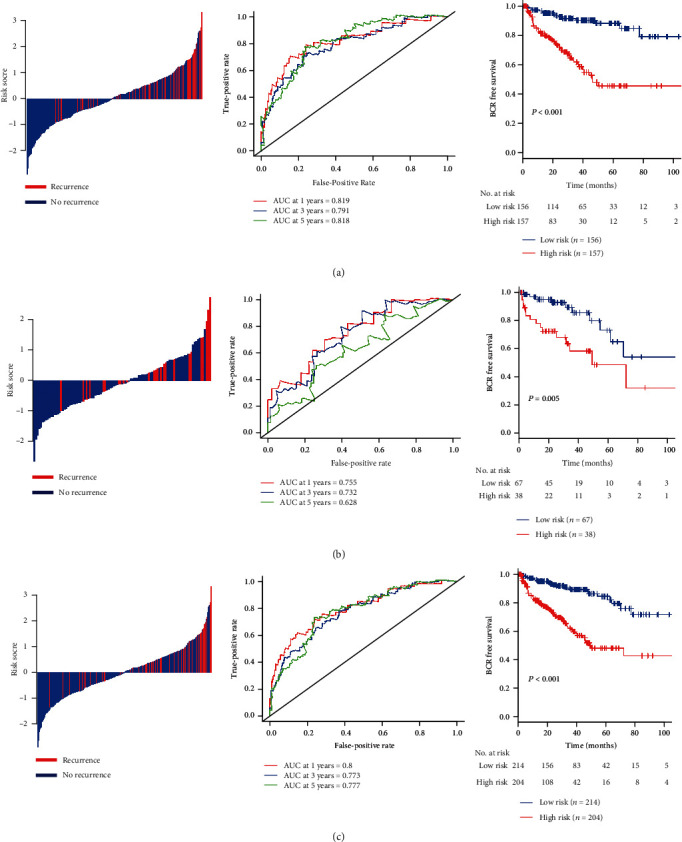
Distribution of BCR risk score, time-dependent ROC curves at 1, 3, and 5 years and Kaplan-Meier survival curves between patients in low and high BCR risk groups in training set (a), internal validation set (b), and entire TCGA cohort (c).

Based on the median risk score (value = 15.6), patients in discovery set were divided into low-risk group (*N* = 156) and high-risk group (*N* = 157). The distribution of BCR status and risk scores was illustrated in Figure 2(a) (left panel), and it suggested that patients with lower risk scores generally had better BCR free survival than those patients with higher risk scores. Time-dependent ROC analyses at 1 year, 3 years, and 5 years revealed a good performance of CSC gene signature (Figure 2(a), middle panel). The BCR free survival rates for patients in low-risk group were 96.6% at 1 year, 90.4% at 3 years, and 88.5% at 5 years, compared with 81.4%, 62.0%, and 45.7% in patients in high-risk group, respectively (HR: 4.91, 95% CI: 2.75–8.76, *P* < 0.001, Figure 2(a), right panel).

According to the same cutoff value determined in discovery set, the same analyses were further performed in the internal validation set. The 1 year, 3-year, and 5-year BCR free survival rates were 94.9%, 92.7%, and 73.2% for the low-risk group and 78.0%, 72.5%, and 48.6% for the high-risk group (HR: 2.99, 95% CI: 1.34–6.70, *P* = 0.005, Figure 2(b)).

In the entire TCGA cohort, CSC gene-based signature yielded similar results (Figure 2(c)). This CSC gene-based classifier can divide PCa patients into low- and high-risk groups with notably different BCR free survival. In addition, the signature showed the highest prognostic accuracy at one year after resection.

### 3.3. Prognostic Performance and Clinical Use of the CSC Gene-Based Signature in Predicting Early BCR

**Figure 3:**
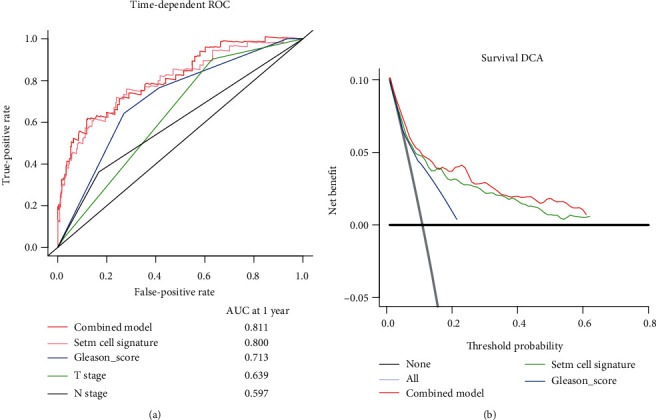
Time-dependent ROC curves at 1 year of the CSC gene signature, Gleason score, T stage, and N stage (a) in the entire TCGA database; decision curve analysis at 1 year for the CSC gene signature, Gleason score, and the combined model (b). The *y*-axis measures the net benefit.

To verify that CSC gene-based signature performs more accurately than clinicopathological features in predicting early BCR, time-dependent ROC was used in the entire cohort, and the result showed that the CSC gene-based signature (AUC = 0.800) had significantly higher prognostic accuracy than Gleason score (AUC = 0.713), T stage (AUC = 0.639), and N stage (AUC = 0.597) at 1 year (Figure 3(a)). The decision curve analysis at 1 year was further used to compare the value of clinical use between CSC gene-based signature and Gleason score. It suggested that using the CSC gene-based signature to predict early BCR added more benefit than either the treat-all-patients scheme or the treat-none scheme, while the Gleason score brought significantly less benefit (Figure 3(b)).

### 3.4. Development of a Nomogram for Predicting Early BCR of PCa Patients

**Figure 4:**
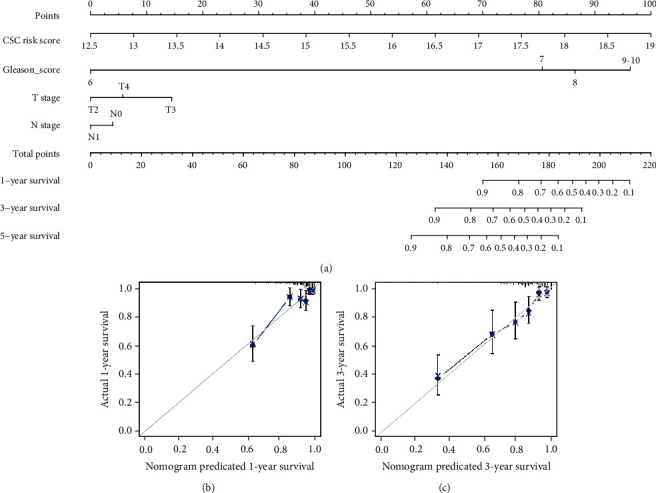
(a) The developed nomogram predicting the risk of BCR at 1, 3, and 5 years in PCa patients. (b) Calibration curves of the nomogram to predict BCR at 1 year. (c) Calibration curves of the nomogram to predict BCR at 3 years.

To provide a quantitative method for clinician to predict the risk of early BCR in PCa, we constructed a nomogram based on CSC gene-based signature, Gleason score, T stage, and N stage (Figure 4(a)). By summarizing the points assigned to each variable being indicated at the top of the scale, we can calculate the total points for each patient. Based on the lowest scale, the total points will be converted to the predicted 1-, 3-, and 5-year rate of BCR free survival for every patient. The developed nomogram showed high predictive ability with a C-index of 0.773. Further calibration curves of this nomogram revealed negligible deviations from the reference line and no need of recalibration (Figures 4(b) (1 year) and 4(c) (3years)).

## 4. Discussion

To be noted, early BCR after initial surgery accounted for almost half of all tumor BCR, suggesting that potential micrometastases and inherent heterogeneity may be essential factors in promoting tumor relapse. Postoperative BCR, ascribed to PCa cell dissemination, is closely related to oncological outcomes, which is dominantly assessed by Gleason score and TNM staging system. However, the prognosis of PCa patients with similar clinicopathological features is always varying considerably because of the innate difference of genetic and epigenetic backgrounds. Early BCR was usually considered as an indicator for emergence of tumor recurrence metastasis and PCa patients with early BCR always suffering from strikingly poorer long-term survival than those without early BCR, in spite of the notable progress of adjuvant treatment. Construction of a reliable predictive model for the detection of early BCR would compensate for the deficiency of Gleason score and TNM classification, thereby facilitating the design of more efficient and individualized postoperative therapeutic strategies.

Recently, mRNA expression-based signature has been widely studied and developed to predict recurrence for PCa patients. However, limited robust classifiers have been constructed to detect early BCR and no previous researchers attempted to identify early BCR-associated CSC genes and develop a CSC gene set-based signature. In 2018, Abou-Ouf et al. established a multigene-based signature for predicting PCa BCR but achieving a weak prognostic accuracy (AUC = 0.65) [20]. In this study, a novel prognostic classifier based on thirteen CSC-associated genes was constructed to improve the prediction of early BCR for PCa after primary surgery. By using this CSC gene signature, patients in the discovery set can be divided into two risk groups with notably different BCR free survival. Besides, it was successfully validated in the internal validation set separated from TCGA cohort. Further, the survival ROC and survival DCA at 1 year indicated that this CSC gene-based signature has considerable higher prognostic accuracy and clinical value in predicting BCR within the first year after primary resection than Gleason score and tumor stage. Therefore, our study identified a thirteen-CSC gene set-based signature that could help to identify patients at high risk of early BCR and guide individualized postoperative treatment of PCa patients, which is credible to be applied to clinic.

Previous studies found that there is a subset of cells in the PCa tissue retaining the capacity of tumor initiation and long-term tumor propagation, suggesting the fundamental role of CSC in PCa [21]. A total of thirteen CSC-associated genes were selected in this study, and all of the genes included in the CSC gene-based signature have been experimentally confirmed to be linked with cancer and of them including CTNNBIP1, FDZ5, LATS2, NAMPT, GREM1, PRKACB, and WNT4 have been proved to have an oncogenic or prognostic role in PCa. CTNNBIP1 is a *β*-catenin-binding protein that inhibits the canonical Wnt/*β*-catenin signaling pathway. Zhuo et al. revealed that the N-terminal helical domain of CTNNBIP1 had a pronouncedly inhibitory effect on *β*-catenin binding to AR proteins [22]. FDZ5 is a member of “frizzled” gene family and is believed to be the receptor for the Wnt5A ligand. Previous studies suggested that FDZ5 involved in mediating the proapoptotic and antiproliferative effects of WNT5A in prostate cancer [23]. GREM1 expression is found in many cancers and is thought to play important roles in regulative cell proliferation and survival of PCa [24]. LATS2 is a tumor suppressor that is homolog of Drosophila warts/lats. LAST2 has been found as a novel AR-interacting protein, playing an essential role in AR-mediated transcription and contributing to the progression of PCa [25]. NAMPT is the rate-limiting enzyme in the NAD+ salvage pathway, and NAMPT activity is required to support fatty acid and lipid synthesis [26]. PRKACB is a catalytic subunit of cAMP-dependent protein kinase which is known to activate the androgen receptor. PRKACB expression has been established as a predictive marker for response to radiation and chemotherapy [27]. WNT4 is one of the family members of WNT, and the expression of WNT4 in PCa has been elucidated as an important factor in both embryonic development and tumor suppression [28]. As for the rest six genes, further clinical and basic research should be performed to reveal the role in PCa.

Though this is the first study investigating the prognostic role of CSC-associated genes and successfully developing a CSC-associated gene set-based signature in PCa, some limitations are inevitable in our study. Firstly, only public-available RNA-sequencing dataset was used, and no external validation was conducted. Furthermore, the information of the margin status and postoperative treatment was not available in this study. Finally, the potential molecular mechanisms of the identified CSC-associated genes contributing early BCR of PCa are still needed to be further investigated.

In conclusion, we successfully construct a robust CSC-associated gene set-based signature that can reliably predict the emergence of early BCR postoperatively in PCa. A prospective clinical trial aiming to validate the prognostic role of the developed the signature should be conducted.

## Figures and Tables

**Table 1:** Basic clinicopathological features of PCa patients in early BCR and long-term survival groups before and after PS matching in the discovery set.

Variables	Discovery set					
	Before matching			After matching		
	Long-term survival (%)	Early BCR (%)	*P*	Long-term survival (%)	Early BCR (%)	*P*
Age (mean, IQR)	60.6 (56.0-66.0)	62.6 (59.0-66.0)	0.153	62.43 (57.0-68.0)	62.43 (58.0-66.0)	1
Gleason score			<0.001			1
6	5 (4.9)	0 (0.0)		0 (0.0)	0 (0.0)	
7	63 (61.2)	8 (25.8)		6 (26.1)	6 (26.1)	
8	12 (12.6)	4 (12.9)		4 (17.4)	4 (17.4)	
9-10	22 (21.4)	19 (61.3)		13 (56.5)	13 (56.5)	
T stage			0.020			1
T2	40 (38.8)	3 (9.7)		3 (13.0)	3 (13.0)	
T3	59 (57.3)	27 (87.1)		20 (87.0)	20 (87.0)	
T4	4 (3.9)	1 (3.2)		0 (0.0)	0 (0.0)	
N stage			0.008			1
N0	91 (88.3)	22 (71.0)		17 (73.9)	17 (73.9)	
N1	12 (11.7)	9 (29.0)		6 (26.1)	6 (26.1)	
Total	103 (100)	31 (100)		23 (100)	23 (100)	

## Data Availability

Source data and reagents are available from the corresponding author upon reasonable request.

## Supplementary Materials

Supplementary materials The detailed information of fold change and significance of these sixteen genes are shown in Table S1.
