# Supplementary material for: Construction and Validation of a Robust Cancer Stem Cell-Associated Gene Set-Based Signature to Predict Early Biochemical Recurrence in Prostate Cancer
Source: Dis Markers. 2020 Oct 9;2020:8860788. doi: 10.1155/2020/8860788 (PMC7569422; doi:10.1155/2020/8860788)
Supplement: Supplementary materials — The detailed information of fold change and significance of these sixteen genes are shown in Table S1. [file 8860788.f1.docx]

| Gene symbol | logFC | AveExpr | t | P.Value | B |
| --- | --- | --- | --- | --- | --- |
| RBPJL | 0.722296 | 0.498152 | 3.200552 | 0.002481 | -2.29199 |
| CTNNBIP1 | 0.351704 | 10.49503 | 2.865365 | 0.006249 | -3.1489 |
| STK36 | 0.421791 | 9.194665 | 2.860847 | 0.006325 | -3.16001 |
| PRKACB | -0.55257 | 10.57022 | -2.79118 | 0.00761 | -3.32978 |
| BMP8B | 0.72547 | 5.537057 | 2.683152 | 0.010087 | -3.58711 |
| BOD1 | 0.2686 | 9.701187 | 2.599756 | 0.012484 | -3.7807 |
| NAMPT | -0.49911 | 11.18348 | -2.322 | 0.024694 | -4.39189 |
| IFNG | -0.7557 | 1.350278 | -2.31629 | 0.025031 | -4.4039 |
| LRP2 | 0.785857 | 2.484589 | 2.297025 | 0.026198 | -4.44422 |
| WNT4 | 0.439783 | 7.436196 | 2.278419 | 0.027371 | -4.48291 |
| SEL1L | -0.40509 | 12.07557 | -2.24247 | 0.029771 | -4.55694 |
| LATS2 | -0.40661 | 9.227693 | -2.21127 | 0.032004 | -4.62044 |
| FZD5 | -0.52773 | 9.983809 | -2.1997 | 0.032869 | -4.64381 |
| GREM1 | 1.114596 | 8.464433 | 2.173997 | 0.034864 | -4.69535 |
| IL17B | 0.593704 | 2.832022 | 2.107672 | 0.040515 | -4.82607 |
| TCF15 | 0.594552 | 2.146407 | 2.060044 | 0.045052 | -4.91789 |

Table S1. Detailed information of sixteen gene identified from discovery series in TCGA
